# Effect of Remote Peer-Counsellor- delivered Behavioral Activation and Peer-support for Antenatal Depression on Gestational Age at Delivery: a single-blind, randomized control trial

**DOI:** 10.1186/s13063-023-07077-7

**Published:** 2023-03-30

**Authors:** Kathleen H. Chaput, Makayla Freeman, Carly McMorris, Amy Metcalfe, Emily E. Cameron, James Jung, Suzanne Tough, Laurel M. Hicks, Sona Dimidjian, Lianne M. Tomfohr-Madsen

**Affiliations:** 1 Department of Obstetrics and Gynecology, Cumming School of Medicine, University of Calgary, 2500 University Drive, NW, Calgary, AB T2N 1N4 Canada; 2 University of British Columbia, Vancouver, Canada; 3 University of Calgary, Calgary, Canada; 4 University of Manitoba, Winnipeg, Canada; 5 University of Colorado, Boulder, USA

**Keywords:** Depression, Pregnancy, Peer counselling, Behavioural activation, Peer support, Preterm birth, RCT

## Abstract

**Background:**

Antenatal depression (AD) is the most common complication of pregnancy in developed countries and increases the risk of preterm birth (PTB). Many pregnant individuals with AD do not obtain treatment due in part to risks associated with antidepressant medications, the expense and wait times for psychological services, and perceived stigma. Accessible and timely treatment of antenatal depression is crucial to minimize foetal impacts and associated long-term child health outcomes. Previous studies show that behavioural activation and peer support are promising avenues of treatment for perinatal depression. Additionally, remote and paraprofessional counselling interventions show promise as more accessible, sustainable, and cost-effective treatment avenues than traditional psychological services. The primary aim of this trial is to test the effectiveness of a remote, behavioural activation and peer support intervention, administered by trained peer para-professionals, for increasing gestational age at delivery among those with antenatal depression. The secondary aims are to evaluate the effectiveness for treating AD prior to delivery, with persistence into the postpartum; improving anxiety symptoms; and improving parenting self-efficacy compared to controls.

**Methods:**

A two-arm, single-blinded, parallel groups randomized controlled trial (RCT) with repeated measures will be conducted. Participants scoring >10 on the Edinburgh Postnatal Depression Scale will be recruited from the larger P3 cohort and invited to enroll. Assessments will be conducted prior to 27 weeks’ gestation at trial intake (T1), post-intervention, prior to delivery (T2), 5–6 months postpartum (T3), and 11–12 months postpartum (T4) and will include self-report questionnaires and linked medical records.

**Discussion:**

Our remote, peer paraprofessional-delivered behavioural activation plus peer support intervention has the potential to successfully reduce symptoms of AD, which may in turn decrease the risk of PTB and subsequent health impacts. The current trial builds on previous findings and uses a patient-oriented approach to address priorities for patient care and to provide a cost-effective, accessible, and evidence-based treatment to pregnant individuals with AD.

**Trial registration:**

International Standard Randomised Controlled Trial Number (ISRCTN) registry (ISRCTN51098220) ISRCTN51098220. Registered on April 7, 2022.

**Supplementary Information:**

The online version contains supplementary material available at 10.1186/s13063-023-07077-7.

## Administrative information

Note: the numbers in curly brackets in this protocol refer to SPIRIT checklist item numbers. The order of the items has been modified to group similar items (see http://www.equator-network.org/reporting-guidelines/spirit-2013-statement-defining-standard-protocol-items-for-clinical-trials/).

**Table:** 

Title {1}	Effect of a remote peer-administered behavioural activation and peer-support intervention for antenatal depression on gestational age at delivery: a single-blind, randomized control trial
Trial registration {2a and 2b}.	The trial was registered in the International Standard Randomised Controlled Trial Number (ISRCTN) registry (ISRCTN51098220) on April 7, 2022.
Protocol version {3}	November 8, 2022 (Version # 5)
Funding {4}	This work was supported by a joint operating grant from the Calgary Health Trust, and the Alberta Children’s Hospital Foundation
Author details {5a}	Kathleen H. Chaput, PhD, University of Calgary Makayla Freeman, University of British Columbia Carly McMorris, PhD, University of Calgary Amy Metcalfe, PhD, University of Calgary Emily E. Cameron, PhD, University of Manitoba James Jung, HBSc, University of Calgary Suzanne C. Tough, PhD University of Calgary Laurel M. Hicks, PhD, University of Colorado Sona Dimidjian, PhD, University of Colorado Lianne Tomfohr-Madsen, PhD, University of Calgary
Name and contact information for the trial sponsor {5b}	Kathleen H. Chaput Department of Obstetrics and Gynecology 434, North tower, Foothills Medical Centre, 1403 29^th^ Street, NW, Calgary, AB, T2N 2T9
Role of sponsor {5c}	This funding source had no role in the design of this study and will not have any role during its execution, analyses, interpretation of the data, or decision to submit results.

## Introduction

### Background and rationale {6a}

Antenatal depression (AD) is common, affecting approximately 15% of pregnant individuals, and is a major risk factor for conditions linked to neonatal intensive care unit (NICU) admission, including preterm birth (PTB), small for gestational age (SGA), and low birth weight (LBW) [1–3]. A recent meta-analysis showed that untreated depression is associated with significantly increased odds of preterm birth (OR=1.56) and low birth weight (OR=1.96) [4]. Further impacts of untreated depression in pregnancy include short- and long-term negative effects on offspring physiological, behavioural, social, and cognitive outcomes [5]. Each case of antenatal depression including child-related costs is estimated at $129,000, or £74,000 translating to a cost of $9.6 billion in Canada or £8.1 billion in the UK every year [6]. Treatment of antenatal depression is therefore crucial to both maternal*^1^ and child health, and to reduce strain on the healthcare system. To minimize foetal impacts and associated long-term child health outcomes, accessible treatments that are effective within the short window of pregnancy are needed.

While pharmacologic treatment represents one effective and relatively fast avenue for treating AD, the evidence remains unclear about exposure to the most common form of pharmacological depression treatment (selective serotonin reuptake inhibitors, SSRIs) and some studies have identified an association with increased risk of preterm birth above and beyond the increased risk associated with depression [7]. Previous research has also found that pregnancy is a major determinant of antidepressant medication cessation [8], and that therapy-based depression treatments are the documented preference of the perinatal population [9].

Several effective, evidence-based therapy treatments exist for AD [10–14]. Preliminary research shows that psychological interventions delivered in pregnancy are associated with reductions with PTB in high-risk populations, and the mechanisms appear to be through improvements in psychological symptom profiles during pregnancy [15–17]. Behavioural activation (BA) is one option that shows promise for effectiveness in treating depression and anxiety during pregnancy [11, 18]. BA is a brief, structured treatment for depression that aims to increase rewarding experiences in a client’s life by increasing activation [11, 18]. However, due to the high costs associated with private psychological services, long wait times for subsidized services, and perceived stigma associated with accessing psychotherapy during pregnancy, fewer than 20% of those with AD obtain treatment [19].

Remotely administered (i.e., online, phone) and paraprofessional counselling interventions offer a potential avenue to increase the accessibility and sustainability of effective AD treatment in Canada, through reduced costs to the patient, and to the system [20]. Evidence supports that effective therapeutic relationships can be established in remote (telephone and internet) counselling sessions [21]. Additionally, therapy that is administered remotely (i.e. via telephone, or video-conference) has been identified as a preferred means of providing treatment and support among depressed individuals in pregnancy, as it is flexible, private, accessible and reduces the stigma associated with seeking treatment [21]. It is also more accessible than traditional treatment options for those living in rural and remote areas. Paraprofessional counselling interventions (psychological therapy administered by a trained lay-person) are highly cost-effective compared with counselling offered by psychologists alone, have been effective for treating depression and anxiety in the general population [20], and have shown sustainability up to 12 months post-trial [22–24].

Recent studies have also highlighted that social and peer support during pregnancy has an important role in reducing the risk of postpartum depression and anxiety, and clinical trials have demonstrated the effectiveness of peer support for preventing postpartum depression [25–28]. Furthermore, in our patient-oriented pilot study of a telephone-based counselling intervention, we found that pregnant individuals at high risk of postpartum depression articulated the need for social and emotional support beyond four provided antenatal therapy sessions and into the early postpartum [29]. This feedback indicates that a combination of therapy and peer support may be a desirable and effective adjuvant to therapy for AD. Results of our pilot testing indicated that peer counsellors may also perceive benefits to their own mental health and well-being as a result of assisting other depressed mothers*.

The current study will test the effectiveness of a remote behavioural activation plus informal ongoing peer-support intervention, administered by trained peer paraprofessional counsellors, for increasing gestational age at delivery and decreasing symptoms of AD, in a randomized controlled trial (RCT). This RCT is nested in the larger Prediction, Prevention and Interventions for Preterm Birth (P3) cohort study in Alberta Canada. The remote- and peer-delivered format of our intervention provides a cost-effective mechanism to increase access to evidence-based treatment for AD, while reducing stigma and responding to patient-articulated needs for more continuous and social-oriented support than can be obtained through traditional psychological therapy. Further, the prevalence of AD and anxiety symptoms increased dramatically during the global COVID-19 pandemic [30–32] highlighting an increasing need for remote interventions for depression during pregnancy, which might also be addressed through our study. We will also conduct qualitative exit interviews with peer counsellors to determine whether the experiences of providing support has had any positive or negative impacts on them personally, and garner feedback on their experiences and perceptions about potential areas where the intervention and delivery process might be improved.

The P3 study will recruit 4000 Albertans during pregnancy and follow them to 12 months postpartum, and aims to identify predictors of preterm birth, identify effective interventions for preterm birth, and improve the care and outcomes for preterm infants and their families. Questionnaire data will be collected before 36 weeks’ gestation (T1), and at 5–6 months (T3) and 11–12 months (T4) postpartum, and be linked with electronic health records. The current study will also involve a questionnaire at intervention completion (T2) to assess immediate post-intervention depression scores.

### Objectives {7}

The primary aim of this study is to conduct an RCT to determine the effectiveness of remote, peer-delivered behavioural activation (BA) therapy, plus ongoing informal peer-support intervention to individuals with AD in reducing preterm birth. We hypothesize that our intervention will be associated with an increase in gestational age at delivery compared to the standard of care.

The secondary aims of this trial are to determine whether the peer-administered intervention will be effective for treating AD prior to delivery, with persistence to 6 and 12 months postpartum, improving anxiety symptoms compared to controls.

We hypothesize that participants in the intervention group will have:

- Significantly greater reduction in depression symptoms from T1 to T2, and significantly lower odds of depression at T2, T3 and T4 compared to controls
- Significantly greater reduction in anxiety symptoms from T1 to T2, and significantly lower odds of depression at T2, T3 and T4 compared to controls

We will also qualitatively explore the lived experiences of peer counsellors in delivering the trial intervention. We hypothesize that experiences providing the intervention to peers will be perceived as positive and that peer counsellors may identify perceived benefits to their own mental well-being.

### Trial design {8}

A trial within cohort design will be conducted [33]. A two-arm, single-blinded, parallel groups superiority design RCT with repeated measures will be used to evaluate the impact of remote (phone and/or online), peer paraprofessional-delivered BA and peer support on gestational length, compared to standard-of-care (SOC), among individuals with antenatal depression. Assessments will be conducted prior to 27 weeks’ gestation at trial intake (T1), within 1 week of inervention completion and prior to delivery (T2), 5–6 months postpartum (T3), and 11–12 months postpartum (T4). The current study will be conducted in adherence with the University of Calgary’s Conjoint Health Research Ethics Board (CHREB) regulations, and the 1964 Helsinki Declaration, and reported in accordance with CONSORT guidelines.

## Methods: participants, interventions and outcomes

### Study setting {9}

Participants will be recruited from the Calgary Health Region in Calgary, Alberta, Canada through the larger P3 cohort, using general advertising and recruitment through maternity and ultrasound clinics. The Calgary Health Region is situated in Southwestern Alberta and is home to 1.5 million residents from urban and rural settings. The P3 cohort baseline questionnaire will be administered electronically to the full cohort sample using securely hosted Qualtrics® survey software and will include depression screening questions that will serve to identify participants eligible for our nested trial [34].

### Eligibility criteria {10}

#### Inclusion and exclusion criteria

English-speaking pregnant participants over the age of 18, who complete the baseline P3 cohort questionnaire, and score >10 on the Edinburgh Postnatal Depression Scale (EPDS), who reside in the Calgary Health Region and are less than 27 weeks’ gestation (to enable completion of the intervention prior to delivery, and allow us to capture the vast majority of preterm deliveries) will be eligible for inclusion. Participants undergoing no current psychotherapy or pharmaceutical treatment, or who have been on a stable dose of anti-depressant medications (e.g. SSRIs) for 3 months or more and score 10 or more on the EPDS will be eligible. We will exclude anyone who has a lifetime diagnosis of severe psychiatric illness (bipolar disorder, schizophrenia, borderline personality disorder, other psychoses) and/or is engaged in an active treatment regimen for a diagnosed mental illness (counselling; non-SSRI medications) at trial intake.

### Who will take informed consent? {26a}

All prospective participants will complete informed consent electronically to participate in the primary P3 cohort, which will include optional consent to be contacted and screened for inclusion in sub-studies, including this nested trial. Those meeting the AD definition (above) will be invited to complete a brief screening questionnaire for the current trial. Once it is determined that a prospective participant is eligible to participate, they will be contacted by trained research assistants, who will explain the study procedures in full detail, provide the participant with written copies of the consent form, and obtain written informed consent for participation in the nested trial.

#### Screening and enrollment

After informed consent is obtained for the P3 cohort, including consent to be contacted for nested trials, participants meeting the study definition of AD will be identified by research assistants using weekly data queries. For the purposes of this study, AD is defined as a score of 10 or greater on the Edinburgh Postnatal Depression Scale (EPDS), which has been validated to measure major depressive disorder against diagnostic interviews (sensitivity 87.7%; specificity 90.9%) in pregnant individuals [34, 35]. All participants with AD will be invited to join this nested trial. Participants will be contacted by trained research assistants, provided with study information, and invited to enrol in the current study.

Written informed consent will be obtained in addition to the P3 cohort consent. Research assistants will screen participants further for eligibility and included participants will be randomized to receive the intervention or standard of care (control). Randomization will be conducted using computer-generated, block-randomization into an intervention group and a control group (receiving standard of care).

### Additional consent provisions for collection and use of participant data and biological specimens {26b}

Not applicable, as there are no ancillary studies using participant data or biological specimens.

## Interventions

### Explanation for the choice of comparators {6b}

An SOC control group will be used as a comparator, as it would be unethical to deny care to any person that is identified as being depressed, once screened for inclusion in our study. The standard of care for depression in Alberta includes provision of resources to seek mental health care, in the form of telephone numbers and the Alberta Health Services Website. Any care is then self-initiated by the participant. This comparison will allow for the best estimate of the potential population impact of our study intervention in Alberta.

### Intervention description {11a}

The intervention has been pilot tested with pregnant individuals and is comprised of a BA therapy module, a short workbook, and informal peer support from study entry until 6 weeks postpartum. The intervention will be primarily administered through the secure and encrypted Zoom for Healthcare by trained peer counsellors (below); peer counsellors will also connect with participants over the telephone as needed (e.g. check-ins) or based on barriers to videoconference (e.g. technical issues, no internet or camera). The counselling module is aligned with the BA treatment manual, adapted from the Alma BA peer-mentorship intervention [36], and tailored to reflect the perinatal context [11, 18]. It includes treatment strategies: mapping, problem-solving, scheduling activities, communicating and activating social supports [18]. Treatment will involve an average of 6–8 structured counselling sessions that are flexible and responsive to individual needs. At the conclusion of every counselling session, the peer counsellors will complete the Session Rating Scale, which solicits feedback from the participant about progress and the quality of the therapeutic relationship, and has been shown to improve participant fidelity as well as perceived therapeutic benefit [37–39]. The workbook content compliments the BA treatment strategies and was developed using patient-oriented methods by members of our team. It includes simple activities that serve as complementary tools for activity scheduling, identifying prenatal and anticipating postnatal stressors, and identifying and activating social supports. It also serves as a print resource that can be consulted between sessions and into the postpartum (and beyond), which was a patient-identified preference in pilot research [29]. Completion of the activities is not required to attend the sessions; participants will be guided through the treatment strategies by the peer counsellors during the sessions. The BA therapy will also be supplemented by informal unstructured peer support, including text and telephone contact between participants and peer counsellors from study enrollment through 6-weeks postpartum. Intervention-pilot results showed the intervention and remote delivery is feasible, the content is acceptable and useful to patients, when administered by a psychologist [29]. Qualitative evaluation showed that participants identified improved parenting confidence in the early postpartum and found the intervention helpful with mitigating stress and improving coping skills [29]. Participants also identified a need for more flexibility in the intervention delivery and a desire for more informal support and ongoing contact after birth that psychologists were unable to provide [29].

Peer counsellors, defined as individuals with previous lived experience of antenatal or postpartum depression, who are stable and recovered, will be recruited from Calgary, Alberta using social media and advertising on post-secondary campuses. Interested peers will be screened for eligibility and suitability, following provision of a separate informed consent to research assistants, the criteria for which include: completion of a minimum of some post-secondary education, demonstrated basic understanding of mental health, and openness to evidence-based treatments for depression. After providing informed consent, peer counsellors will complete a 4-week training program adapted from a previous pilot-tested BA training program. BA training consists of weekly asynchronous learning through online training videos and written content, weekly practice of skills as guided by the online content, and weekly virtual group meetings with other peer counsellors to review training content. These meetings will be facilitated by a supervising clinical psychologist certified in the Alma BA program and graduate trainees in clinical psychology (peer supervisors). Weekly training commitment is estimated at 3 h total on average, with options for additional training support as requested or deemed necessary. The weekly virtual meetings will consist of brief didactic review of the weekly content led by supervisors (i.e., graduate students or clinical psychologist), opportunity for questions and discussions of the weekly content, and live skill practice with other peer counsellors and supervisors. Supervisors will have a minimum of one hour of virtual face-to-face contact with peer counsellors (e.g. during weekly virtual group sessions or one-on-one check-in if absent) during the training to provide feedback on skill development and connect with peers requiring additional training support. Peer counsellors will engage in mock-counselling role plays with trainers and other peers (observed by trainers) throughout training to ensure their proficiency in applying the therapeutic skillset developed from the training modules. Trainers will meet following training sessions to discuss the ongoing evaluation of peer counsellors using a standardized scoring system (Additional file 1: Appendix A). Training will also be provided on how and when to initiate a structured referral pathway for crises that can arise (i.e., suicidality, indications of intent to harm self or others, disclosure of immediate emergency), requiring direction and/or contact with the supervising clinician or emergency services. Additional session components, such as weekly administration of the session-wise measures (Outcome Rating Scale, post-session fidelity checklist) [40] and technology considerations for virtual delivery, will be included in the formal training. Finally, the supervising psychologist will provide peers with a “competency approval” when they are ready to move forward to administer the trial intervention, based on a final assessment of their BA knowledge, competence and confidence with applying the intervention materials. This recruitment and training method has been successfully pilot tested and resulted in a recruitment of double the needed sample size in 2 weeks, of which 83.3% were eligible and successfully completed training [29].

**Fig. 1:**
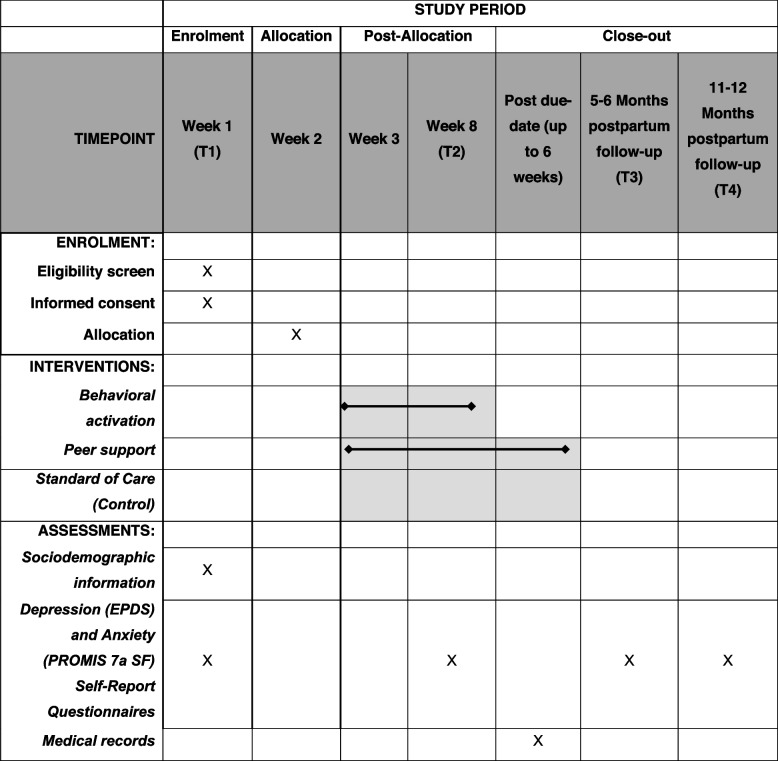
SPIRIT schedule of enrolment, intervention, and assessments

Once the peer counsellors have obtained their competency approval, they will be paired with a single randomized participant and will establish contact with them to initiate and administer the intervention. The peer-delivered intervention will be conducted through Zoom for Healthcare and telephone contact between the peer counsellors and participants. Zoom sessions will be recorded for the purposes of supervision, which is intended for both clinical oversight and continued refinement of peer counsellor’s competencies and therapeutic skillset. In addition to between 6 and 8 structured BA therapy sessions, peer counsellors will also provide informal and unstructured peer support via telephone and/or text. This will include both participant-initiated and peer counsellor-initiated check-ins, sharing of information, and peer guidance. The peer support component will be available to participants up to 6 weeks postpartum, for a total possible length of time of 12 weeks. Peer counsellors may opt to be paired with subsequent participants following completion of the intervention and peer support components. All peer counsellors will receive weekly mandatory clinical supervision via 60-min remote group meetings provided by a clinical psychology graduate trainee, and additional individual supervision meetings as needed, to ensure patient safety and treatment fidelity, and peer counsellor adherence to the protocol. All trainee peer supervisors will be supervised by a registered clinical psychologist through regular virtual group supervision and one-on-one supervision as needed. Fidelity will be addressed with the weekly supervision sessions with the peer counsellors. During the weekly supervision sessions, it will be emphasized that external resources outside of what we provide them cannot be recommended. We will also request that peer counsellors keep an informal record of peer support that they deliver, which will be reviewed during supervision. Peer counsellors will be offered a stipend of $250 for each intervention cycle administered to a single participant, to acknowledge their time and contribution to the study. Following their final intervention cycle in the current study, peers will be invited to complete a qualitative individual in-depth interview, conducted by a trained research assistant under the supervision of the PIs (Chaput, Tomfohr-Madsen). Interviews will elicit reflective conversation around the experiences of peers in delivering the intervention, and any perceived positive or negative impacts the experience may have on their well-being. We will also explore their thoughts and opinions on strengths and weaknesses of the intervention and its delivery (including the training experience) and whether they have suggestions for improvement in the future.

The control group will receive standard of care, including receiving routine obstetric care and referral to community mental health resources, and will also complete all study assessments.

### Criteria for discontinuing or modifying allocated interventions {11b}

Following randomization, peer counsellors will attempt to reach participants via telephone, text, and email (as necessary). Participants who do not respond after 8–10 attempts to establish contact will no longer receive treatment but will remain in the study. If any response is provided to attempted contact, peer counsellors will continue to reach out to schedule remote-counselling sessions, unless the participant expresses a desire to withdraw from the study or they reach the end of pregnancy. Participants will also no longer receive treatment if they do not complete the intervention before the end of pregnancy. If acute suicidality is indicated, either through the study questionnaires or in weekly remote-counselling sessions, the participant will no longer receive treatment and the clinical team will provide a referral to the appropriate care resources using the established referral pathway.

### Strategies to improve adherence to interventions {11c}

Peer counsellors will schedule weekly remote-counselling sessions in accordance with the participants’ availability and will remind participants of upcoming sessions about 24 h before the session or as decided with the participant. Sections of the self-administered workbook will be assigned for each session and participants will be reminded of the assigned sections. In the case that a participant has not completed the assigned materials before the session, the peer counsellor will guide the participant through the workbook content during the session time. In each session, peer counsellors will inquire as to the amount of assigned materials completed before session and any barriers to completing the BA materials. To ensure peer counsellor adherence to the protocol, peer counsellors will also attend a weekly 90-min clinical supervision with a member of the clinical team.

### Relevant concomitant care permitted or prohibited during the trial {11d}

Information on concomitant care will be gathered and tracked throughout the study to ensure that participants receiving care are evenly disbursed between groups. Inclusion will be restricted to individuals who meet the eligibility criteria and who do not have any current active treatment regimen for a diagnosed mental illness (e.g. medications and/or counselling), with the exception of participants who have been on a stable dose of anti-depressant medications (e.g. SSRIs) for 3 months or more. Participants receiving other psychological interventions for mental health problems, including depression and anxiety, will be excluded from the study.

### Provisions for post-trial care {30}

As participants in the control group will be offered standard of care, there will be no provision of the intervention post-trial. Standard of care materials, including a list of community resources, will be provided to both the treatment and control groups.

### Outcomes {12}

The primary outcome is gestational age at birth in days, measured using electronic health records, obtained for the P3 Cohort.

Secondary outcomes are:

- Change in symptoms of depression across T1 and T2 between the treatment and control groups, measured as overall score on the EPDS; and lower odds of scoring 13 or greater on the EPDS at T2, T3, and T4.
- Change in symptoms of anxiety across all timepoints (measured by the PROMIS – anxiety 7a-item short form self-report questionnaire [41])

### Participant timeline {13}

The overall participant timeline is illustrated in Fig. 1.

Week 1 (T1): Participants of the P3 Cohort study will complete a baseline assessment during pregnancy (less than 27 weeks GA). Individuals who are eligible for this study will be identified and will be contacted by a member of the study team to be invited to participate. The study team member will outline the study protocols and obtain informed consent.

Week 2: Participants will be randomly assigned, using computer-generated block randomization, into the intervention group or the standard of care control group.

Weeks 3–6: Participants randomized to the intervention group will complete the BA counselling sessions with a peer counsellor via telephone, in addition to regular informal peer-support contact. Participants in the control group will continue to receive standard of care.

Week 8–10 (T2): At 34 to 36 weeks gestation, after the completion of the intervention, all participants will be invited to complete an electronic P3 Cohort questionnaire.

Post-due-date (up to 6 weeks postpartum): After childbirth, information about gestational age at delivery will be collected for the P3 cohort via linkage to the participant medical record. Participants will continue to have informal peer-support (social support, advice, information sharing) contact with peer counsellors via text and telephone, up to 6 weeks postpartum.

Five to 6 months postpartum (T3): All participants will be invited to complete an electronic questionnaire as part of their participation in the P3 Cohort.

Eleven to 12 months postpartum (T4): All participants will be invited to complete an electronic questionnaire as part of their participation in the P3 Cohort.

### Sample size {14}

This sample size of 246 participants (123 per group) is adequate to detect a minimally clinically significant change of 3 days in mean gestational age with 80% power at an α level of 5%. Based on a 2-sided *T*-test statistic and an α level of 5%, we will have 90% power to detect a minimally clinically significant difference of 25% in EPDS scores at intervention completion (T1), with a sample size of 214 participants [42]. We have inflated our estimated sample size by a factor of 15% (total *N*=246), to account for study attrition. A prior study demonstrated a reduction in EDPS scores up to 56% in a similar intervention during pregnancy [11]. A total of 60–75 peer counsellors will be recruited and trained to provide the intervention to between 1 and 3 participants through the course of the study. Additional peer counsellors will be recruited and trained as needed.

### Recruitment {15}

Participants will be identified and recruited through the P3 Cohort, which will engage participants through ultrasound clinics, outpatient obstetric clinics, obstetrical triage, and advertising in the community. An informational postcard will be shared by clinic staff with potential participants, who will then be able to self-refer by providing their contact information to be contacted by a member of the P3 study team. Online advertising will contain a link to register for more information about the study, and participants will subsequently be contacted by the study team.

## Assignment of interventions: allocation

### Sequence generation {16a}

Participants will be randomized using computer-generated blocked randomization (block size 4) in a 1:1 ratio to the intervention group or the control group. 123 participants will be randomized into each study group, with a total of 246 participants enrolled in the RCT.

### Concealment mechanism {16b}

The randomization list and participant group assignments will be saved in a password-protected file saved on a secure server. Only research assistants on the P3 study team who are not involved in the analysis for the trial, and therefore unblinded, will have access to the file. Participant blinding is not possible with informed consent for a behavioural intervention.

### Implementation {16c}

A senior research coordinator with experience in randomized trials will be unblinded to study allocations. The coordinator will conduct randomization and generate an allocation list, with the support of a biostatistician external to our study team. P3 research assistants will then notify peer counsellors of their assigned participants as they enrol and provide necessary contact information.

## Assignment of interventions: blinding

### Who will be blinded {17a}

The study investigators will be blinded. The participants, peer counsellors, and clinical supervisors will not be blinded to the study. Lastly, the research assistant responsible for randomization and study coordination will not be blinded.

### Procedure for unblinding if needed {17b}

Not applicable, as participants, peer counsellors, and clinical supervisors will not be blinded.

## Data collection and management

### Plans for assessment and collection of outcomes {18a}

#### Primary outcome

*Gestational age at delivery* will be collected from linked hospital records. Participants will be asked upon enrollment to the P3 cohort to consent to data linkage and hospital chart review to provincial health care databases for themselves and their infant. Standardized medical information, including gestational age at delivery, NICU admission, length of stay, and hospital readmission, will be collected.

#### Secondary outcomes

*Symptoms of depression* will be measured using the Edinburgh Postnatal Depression Scale (EPDS). The EPDS is a 10-item questionnaire that will be administered at less than 27 weeks gestation (T1), 34–36 weeks’ gestation (T2, post-intervention), 5–6 months postpartum (T3), and 11–12 months postpartum (T4). The EPDS has been validated to measure major depressive disorder against diagnostic interviews in pregnant individuals and is a valid and reliable measure of symptoms of depression during the perinatal period [34, 35, 43].

#### Symptoms of anxiety

*Symptoms of anxiety* will be measured using the PROMIS anxiety 7a-item short form [41]. The PROMIS anxiety 7a-item short form is a questionnaire that provides a valid and reliable measure of symptoms of anxiety, including when administered with a perinatal population [34, 41]. The PROMIS anxiety 7a-item short form will be administered at T1, T2, T3, and T4.

#### Lived experiences of delivering the peer-intervention

*Lived experiences of delivering the peer-intervention* will be measured using thematic analysis of qualitative transcript data. Individual in-depth interviews will elicit reflective conversation around the experiences of peers in delivering the intervention, and any perceived positive or negative impacts the experience may have on their well-being. We will also explore their thoughts and opinions on strengths and weaknesses of the intervention and its delivery (including the training experience) and whether they have suggestions for improvement in the future (Additional file 2: Appendix B qualitative interview guide)

#### Descriptive measures

*Demographic data* will be collected at baseline (T1) and will include age, work status, education, experiences of everyday discrimination and Hunger Vital Signs*.* Data on medication use will also be collected at study enrollment, as part of the eligibility criteria.

### Plans to promote participant retention and complete follow-up {18b}

Peer counsellors will contact participants to book weekly remote-counselling sessions. Feedback gathered from the P3 Cohort parent advisory council will inform participant retention strategies, including reminders for questionnaires, congratulatory e-cards following the birth of their infant, and regular communication. In our patient-oriented pilot study including over 140 participants, over 80% of the sample was retained over 1 year [29].

### Data management {19}

In accordance with the University of Calgary’s Data Retention Policy, data will be stored on a secure server in compliance with TCPS-II guidelines, to which only the primary investigators and study team will have access.

### Confidentiality {27}

The confidentiality of participant data and identity will be protected in accordance with the University of Calgary ethics guidelines throughout the recruitment, assessment, treatment, and data analysis. All members of the study team, including peer counsellors, will sign a confidentiality and non-disclosure agreement prior to their involvement in the study. Peer counsellors will be required to sign non-disclosure agreements, and instructed to store participant identifying information on a secure password-protected device to which they have sole access. All participant identifying information will be password-protected and stored on secure servers at the University of Calgary, separate from anonymized data.

Participants will be informed of the limits to confidentiality as part of the informed consent process. Participants will also be informed that absolute confidentiality cannot be guaranteed in a remote environment, as information transmitted via the internet or using personal devices (e.g. the participant’s cell phone) may not be completely secure.

### Plans for collection, laboratory evaluation and storage of biological specimens for genetic or molecular analysis in this trial/future use {33}

Not applicable, as no biological specimens will be collected.

## Statistical methods

### Statistical methods for primary and secondary outcomes {20a}

We anticipate that the block randomization will successfully eliminate confounding by covariates, but potential confounding variables will be measured on all questionnaires, and imbalance between intervention and control groups assessed prior to the final analysis. Any imbalance in confounding variables between the groups will be controlled for as appropriate, using cox proportional hazards or logistic regression modelling. Descriptive statistics will be calculated for all participant characteristics that have the potential to act as confounding variables on the primary association between the intervention and gestational age.

Time-to-event analysis will be used to assess change in our primary outcome of gestational age at delivery.

Logistic regression will be used to model the association between the intervention and categorical secondary outcome of depression (scoring 13 or greater on the EPDS), post-intervention, and 6 and 12 months postpartum. Secondary outcomes of difference in continuous scores on the EPDS (depression), PROMIS (anxiety), and parenting self-efficacy scales between intervention and control groups will be modelled using linear regression.

### Interim analyses {21b}

No interim analyses will be conducted due to the low-risk nature of our intervention, and the relatively short period of time in which it is administered.

### Methods for additional analyses (e.g. subgroup analyses) {20b}

Sub-group analysis will be conducted to compare the medication and no-medication groups using the above analysis methods, as a secondary exploration.

The qualitative analysis of peer experiences will be achieved using emergent thematic analysis of verbatim transcript data. Qualitative interviews of peers will take place via Zoom for Healthcare, or in-person in a private room at the University of Calgary (Pandemic guidelines permitting) and will be approximately 1 hour in length. Interviews will be audio recorded and transcribed verbatim, by a certified transcription service. Transcripts will be coded using emergent thematic analysis.

### Methods in analysis to handle protocol non-adherence and any statistical methods to handle missing data {20c}

Analysis will be completed in accordance with an intention-to-treat (ITT) model and will therefore include all participants, regardless of protocol adherence or missing data. A secondary sensitivity analysis will also be conducted including only those who completed the intervention with adherence to the treatment protocol.

### Plans to give access to the full protocol, participant level-data and statistical code {31c}

K. Chaput will be the primary custodian of the data. All de-identified data and analysis code will be made available upon reasonable request following the completion of all primary, secondary and exploratory analyses by the study team.

## Oversight and monitoring

### Composition of the coordinating centre and trial steering committee {5d}

The University of Calgary’s Cumming School of Medicine will be the coordinating centre for the trial. Co-PI Chaput is an early career investigator and perinatal epidemiologist in the departments of Obstetrics and Gynaecology, Community Health Sciences and Paediatrics in the University of Calgary’s Cumming School of Medicine. Her program of research centres on maternal mental health and substance use in pregnancy and the postpartum. Co-PI Tomfohr-Madsen is a clinical psychologist and associate professor of psychology at the University of Calgary. Chaput and Tomfohr-Madsen will lead the study operations and analysis with oversight and input from an experienced interdisciplinary steering committee:

- Carly McMorris, is a Clinical Psychologist in the Werklund School of Education at the University of Calgary, whose research focuses on maternal and child mental health and development. Dr. Amy Metcalfe, epidemiology lead of the P3 cohort, is a perinatal epidemiologist and an Associate Professor in the Departments of Obstetrics & Gynaecology, Community Health Sciences, and Medicine at the University of Calgary. Her research program focuses on the management of high-risk pregnancies.
- Laurel Hicks is a research scientist at the University of Colorado Boulder in the Renee Crown Wellness Institute and a Licensed Clinical Social worker who specialized in perinatal mental health and is an expert in the Alma BA peer-mentorship intervention.
- Sona Dimidjian, University of Colorado, is a Professor of Psychology and Neuroscience and Director of the Renee Crown Wellness Institute at the University of Colorado Boulder. She is the developer of the Alma BA peer intervention.
- Suzanne Tough is an epidemiologist and professor in the department of Paediatrics at the University of Calgary and brings expertise in design and management of longitudinal epidemiologic studies of maternal health, and a vast network for knowledge translation.

The Cumming School of Medicine offers Randomised Trial supports including database management and compliance monitoring. Through memberships in the Alberta Children’s Hospital Research Institute, the Hotchkiss Brain Institute, and the O’Brien Institute for Public Health, the team has access to graduate training studentships, student project funding, methods consultation, communications support, interdisciplinary research rounds, support for online data collection and management, knowledge translation support, and yearly research symposia. UCalgary provides Equity Diversity and Inclusion (EDI) facilitation for hiring and trainee selection, and departmental meeting facilities, administrative support, and research nurses to aid with recruitment.

### Composition of the data monitoring committee, its role and reporting structure {21a}

Data will be monitored by co-PIs Chaput and Tomfohr Madsen, with oversight from the larger P3 Cohort leads (Metcalfe, Leijser, Slater). PIs will meet regularly with research assistants to monitor data integrity, participant follow-up and attrition.

### Adverse event reporting and harms {22}

Peer counsellors will receive regular, weekly clinical supervision from a team of qualified clinical psychology supervisors, with senior oversight from PI Tomfohr-Madsen. Supervision will ensure referral of participants to appropriate care if the need arises and provide guidance for peer counsellors in managing participant concerns, circumstances and clinical needs. Participants will be informed to contact research assistants and/or co-PIs to report potential adverse events, which will then be reported in accordance with University of Calgary Conjoint Health Research Ethics Board (CHREB) guidelines.

### Frequency and plans for auditing trial conduct {23}

Given the short time span of the trial, no regular audits are scheduled a-priori. Trial conduct audits will be performed at the request of the University of Calgary’s CHREB.

### Plans for communicating important protocol amendments to relevant parties (e.g. trial participants, ethical committees) {25}

Should safety needs arise, the approval of any protocol amendments will be sought from CHREB prior to implementation. Consent addendums will then be communicated to all enrolled, active trial participants.

### Dissemination plans {31a}

We will communicate key findings through peer-reviewed publications, conference presentations, and reporting provided to the Maternal Newborn Child and Youth strategic clinical network, Alberta Health Services. Findings will also be disseminated via the P3 cohort dissemination strategy that will include reporting to 4 stakeholder groups: (1) pregnant individuals, new parents and their families; (2) health care professionals caring for pregnant individuals and newborns; (3) health care organizations providing care to pregnant individuals and newborns; and (4) academics.

## Discussion

The current trial builds on previously identified priorities for patient care, identified by new parents, health service professionals, policy makers, and patients, using a patient-oriented approach [3]. Current screening efforts are working to identify more perinatally depressed individuals; however, many patients go undiagnosed, and those who are diagnosed face long wait times and financial barriers to accessing care, which substantially increases their risk of preterm birth, and negative sequelae for their infants. The peer-counsellor model has proven to be a cost-effective alternative to standard psychological care in developing countries and for depression in the general population [20, 44, 45]. Building on this, along with evidence that peer support is a successful model for improving antenatal mental health outcomes in pregnant individuals with depression [26, 27], we hypothesize that a peer counsellor administration of our intervention could provide a cost-effective method of preventing NICU admission in high-risk pregnant individuals in Alberta, providing treatment for antenatal depression in a timely, sustainable and patient-oriented manner. If successful our intervention could improve parental and infant health, and substantially reduce the burden of care currently faced by the Alberta health care system.

## Trial status

The P3 Cohort began recruitment in September of 2021. Recruitment and training of peer counsellors for the current trial is slated to begin in fall 2022, with participant enrollment to begin immediately following the completion of training by the first cohort of peer counsellors, approximately two months later. Our final enrollment is projected to occur in 2024. The trial protocol was last updated in November 2022 (Version 5).

## Supplementary Information

**Additional file 1: Appendix A.** Review of Alma Mentoring for Peers.

**Additional file 2: Appendix B.** Qualitative peer-counsellor exit-interview guide – P3 PAAD Trial.

## Data Availability

Data will be maintained in a secure database, accessible only by study personnel.

## Abbreviations

AD: Antenatal depression
BA: Behavioural activation
EPDS: Edinburgh Postnatal Depression Scale
GA: Gestational age
ITT: Intention to treat
LBW: Low birth weight
PTB: Preterm birth
RCT: Randomized controlled trial
SGA: Small for gestational age
SOC: Standard of care
SSRIs: Selective serotonin reuptake inhibitors
CHREB: Conjoint Health Research Ethics Board

## Footnotes

1 The term Maternal* and mothers* is used throughout this document to denote the health condition of the pregnant parent, and acknowledges that this includes non-female identifying people. It is used in the scientific context to differentiate from parental conditions which can include those experienced by fathers and non-pregnant partners.

## References

[1] Alder J, Fink N, Bitzer J, Hösli I, Holzgreve W (2007). Depression and anxiety during pregnancy: a risk factor for obstetric, fetal and neonatal outcome? A critical review of the literature. J Maternal-Fetal Neonatal Med.

[2] Staneva A, Bogossian F, Pritchard M, Wittkowski A (2015). The effects of maternal depression, anxiety, and perceived stress during pregnancy on preterm birth: A systematic review. Women Birth.

[3] Dadi AF, Miller ER, Bisetegn TA, Mwanri L (2020). Global burden of antenatal depression and its association with adverse birth outcomes: an umbrella review. BMC Public Health.

[4] Jarde A, Morais M, Kingston D, Giallo R, MacQueen GM, Giglia L (2016). Neonatal Outcomes in Women With Untreated Antenatal Depression Compared With Women Without Depression: A Systematic Review and Meta-analysis. JAMA Psychiat.

[5] Gentile S (2017). Untreated depression during pregnancy: Short- and long-term effects in offspring. A systematic review. Neuroscience.

[6] Bauer A, Parsonage M, Knapp M, Iemmi V, Adelaja B, Hogg S (2014). The costs of perinatal mental health problems.

[7] Eke AC, Saccone G, Berghella V (2016). Selective serotonin reuptake inhibitor (SSRI) use during pregnancy and risk of preterm birth: a systematic review and meta-analysis. Bjog-Int J Obstet Gy.

[8] Petersen I, Gilbert RE, Evans SJW, Man S-L, Nazareth I (2011). Pregnancy as a major determinant for discontinuation of antidepressants: an analysis of data from The Health Improvement Network. J Clin Psychiatry.

[9] Battle CL, Salisbury AL, Schofield CA, Ortiz-Hernandez S (2013). Perinatal antidepressant use: Understanding women’s preferences and concerns. J Psychiatr Pract.

[10] Green SM, Frey BN, Donegan E, McCabe RE (2018). Cognitive Behavioral Therapy for Anxiety and Depression During Pregnancy and Beyond: How to Manage Symptoms and Maximize Well-Being.

[11] Dimidjian S, Goodman SH, Sherwood NE, Simon GE, Ludman E, Gallop R (2017). A pragmatic randomized clinical trial of behavioral activation for depressed pregnant women. J Consult Clin Psych.

[12] Loughnan SA, Wallace M, Joubert AE, Haskelberg H, Andrews G, Newby JM (2018). A systematic review of psychological treatments for clinical anxiety during the perinatal period. Arch Womens Ment Health.

[13] Sockol LE (2015). A systematic review of the efficacy of cognitive behavioral therapy for treating and preventing perinatal depression. J Affect Disord.

[14] Sockol LE (2018). A systematic review and meta-analysis of interpersonal psychotherapy for perinatal women. J AFFECT DISORDERS..

[15] Bublitz MH, Nillni Y, Livingston Z, Carpenter M, Salmoirago-Blotcher E (2019). Phone-delivered mindfulness training for pregnant women at risk for preterm birth. J Altern Complement Med.

[16] Goodman SH, Cullum KA, Dimidjian S, River LM, Kim CY (2018). Opening windows of opportunities: Evidence for interventions to prevent or treat depression in pregnant women being associated with changes in offspring’s developmental trajectories of psychopathology risk. Dev Psychopathol.

[17] MacKinnon AL, Madsen JW, Giesbrecht GF, Campbell T, Carlson LE, Dimidjian S, et al. Effects of Mindfulness-Based Cognitive Therapy in Pregnancy on Psychological Distress and Gestational Age: Outcomes of a Randomized Controlled Trial. Mindfulness. 2021.

[18] Martell CR (2010). Behavioral activation for depression a clinician’s guide.

[19] Singla DR, Meltzer-Brody SE, Silver RK, Vigod SN, Kim JJ, La Porte LM (2021). Scaling Up Maternal Mental healthcare by Increasing access to Treatment (SUMMIT) through non-specialist providers and telemedicine: a study protocol for a non-inferiority randomized controlled trial. Trials.

[20] Singla DR, Lawson A, Kohrt BA, Jung JW, Meng Z, Ratjen C (2021). Implementation and effectiveness of nonspecialist-delivered interventions for perinatal mental health in high-income countries: a systematic review and meta-analysis. JAMA Psychiatry.

[21] Lingley-Pottie P, McGrath PJ (2007). Distance therapeutic alliance: The participant’s experience. Adv Nurs Sci.

[22] Armstrong J (2010). How effective are minimally trained/experienced volunteer mental health counsellors? Evaluation of CORE outcome data. Counsel Psychother Res.

[23] Atkins DC, Christensen A (2001). Is Professional Training Worth the Bother? A Review of the Impact of Psychotherapy Training on Client Outcome. Aust Psychol.

[24] Bright JI, Baker KD, Neimeyer RA (1999). Professional and Paraprofessional Group Treatments for Depression: A Comparison of Cognitive-Behavioral and Mutual Support Interventions. J Consult Clin Psych.

[25] Hetherington E, McDonald S, Williamson T, Patten SB, Tough SC (2018). Social support and maternal mental health at 4 months and 1 year postpartum: analysis from the all our families cohort. J Epidemiol Commun H..

[26] Dennis C-L (2010). Postpartum depression peer support: Maternal perceptions from a randomized controlled trial. Int J Nurs Stud.

[27] Dennis C-L (2014). The process of developing and implementing a telephone-based peer support program for postpartum depression: Evidence from two randomized controlled trials. Trials.

[28] Dennis CL, Hodnett E, Kenton L, Weston J, Zupancic J, Stewart DE (2009). Effect of peer support on prevention of postnatal depression among high risk women: multisite randomised controlled trial. BMJ.

[29] Chaput K, Vekved M, MacDonald S, McGrath P, Tough S. Pilot testing recruitment and training of peer-para-professional counsellors for prenatal telephone-based Psychotherapy for antenatal depression. Submitted. 2021.

[30] Lebel C, MacKinnon A, Bagshawe M, Tomfohr-Madsen L, Giesbrecht G (2020). Elevated depression and anxiety symptoms among pregnant individuals during the COVID-19 pandemic. J Affect Disord.

[31] Fryer K, Delgado A, Foti T, Reid CN, Marshall J (2020). Implementation of obstetric telehealth during COVID-19 and beyond. Matern Child Health J.

[32] Nelson GA, Holschuh C (2021). Evaluation of telehealth use in prenatal care for patient and provider satisfaction: a step towards reducing barriers to care. J Nurse Pract.

[33] Kim SYH, Flory J, Relton C (2018). Ethics and practice of trials within cohorts: an emerging pragmatic trial design. Clin Trials.

[34] Tendais I, Costa R, Conde A, Figueiredo B (2014). Screening for Depression and Anxiety Disorders from Pregnancy to Postpartum with the EPDS and STAI. Span J Psychol.

[35] Dennis CL, Hodnett ED (2007). Psychosocial and psychological interventions for treating postpartum depression. Cochrane Db Syst Rev..

[36] Dimidjian, S. Randomized controlled trial of the alma peer mentoring program for pregnant women experiencing depression. Registered Trial Protocol: clinical trials.gov. 2016; NCT02883686

[37] Miller SD, Hubble MA, Chow D, Seidel J (2015). Beyond measures and monitoring: Realizing the potential of feedback-informed treatment. Psychotherapy.

[38] Murphy MG, Rakes S, Harris RM (2020). The psychometric properties of the session rating scale: a narrative review. J Evid Based Soc Work (2019).

[39] Duncan BL, Miller SD, Sparks JA, Claud DA, Reynolds LR, Brown J, Johnson LD (2003). The Session Rating Scale: Preliminary psychometric properties of a “working” alliance measure. J Brief Ther.

[40] Miller SD, Duncan BL, Brown J, Sparks JA, Claud DA (2003). The outcome rating scale: A preliminary study of the reliability, validity, and feasibility of a brief visual analog measure. J Brief Ther.

[41] Schalet BD, Pilkonis PA, Yu L (2016). Clinical validity of PROMIS Depression, Anxiety, and Anger across diverse clinical samples. J Clin Epidemiol.

[42] Rini CK, Dunkel-Schetter C, Wadhwa PD, Sandman CA (1999). Psychological Adaptation and Birth Outcomes: The Role of Personal Resources, Stress, and Sociocultural Context in Pregnancy. Health Psychol.

[43] Gibson J, McKenzie-McHarg K, Shakespeare J, Price J, Gray R (2009). A systematic review of studies validating the Edinburgh postnatal depression scale in antepartum and postpartum women. Acta Psychiatr Scand.

[44] Weobong B, Weiss HA, McDaid D, Singla DR, Hollon SD, Nadkarni A (2017). Sustained effectiveness and cost-effectiveness of the Healthy Activity Programme, a brief psychological treatment for depression delivered by lay counsellors in primary care: 12-month follow-up of a randomised controlled trial. PLoS Med.

[45] Singla DR, Weobong B, Nadkarni A, Chowdhary N, Shinde S, Anand A (2014). Improving the scalability of psychological treatments in developing countries: An evaluation of peer-led therapy quality assessment in Goa, India. Behav Res Ther.

