# Supplementary material for: Effect of Remote Peer-Counsellor- delivered Behavioral Activation and Peer-support for Antenatal Depression on Gestational Age at Delivery: a single-blind, randomized control trial
Source: Trials. 2023 Mar 30;24:240. doi: 10.1186/s13063-023-07077-7 (PMC10061403; doi:10.1186/s13063-023-07077-7)
Supplement: Supplementary file 1 — Additional file 1: Appendix A. Review of Alma Mentoring for Peers. [file 13063_2023_7077_MOESM1_ESM.pdf]

Appendix A
Review of Alma Mentoring for Peers

Trainer Completing Rating: ________________________________ Date of Rating: _______________

Peer Mentor: _____________________________________

Participant Mom ID: ________ Meeting Number:___________ Meeting Date: __________________

PART 1. Alma ENLIVEN Skills				
**ESTABLISHES AND FOLLOWS AN AGENDA**				
a. Peer mentor set an agenda towards the beginning of the meeting	Not Done	Partially Done	Done	
b. Peer mentor developed an agenda that included relevant and specific topics for the meeting (e.g., what is most important for the mentee)	Not Done	Partially Done	Done	
c. Peer mentor followed the agenda unless modifications were agreed upon by mentor and mentee	Not Done	Partially Done	Done	
**NURTURES ACTIVATION**				
a. Peer mentor focused on Alma skills. Non-Alma conversation was minimal unless important for building and sustaining rapport.	Not Done	Partially Done	Done	
**LEARNS TOGETHER AS A TEAM**				
a. Peer mentor asked open-ended questions	Not Done	Partially Done	Done	
b. Peer mentor encouraged mentee to be an active collaborator during the meeting	Not Done	Partially Done	Done	
c. Peer mentor checked with the mentee to learn if there is mutual understanding of the material being presented and mutual agreement with the proposed plans (i.e. mentor did not present Alma in a prescriptive, authoritarian way and did not give unsolicited advice)	Not Done	Partially Done	Done	
d. Peer mentor paced the meeting so that the mentor and mentee are working together as a team (i.e., not too fast or too slow)	Not Done	Partially Done	Done	
e. Peer mentor provided an opportunity to summarize main points or “take home messages” during or at end of meeting	Not Done	Partially Done	Done	
**IS NON-JUDGMENTAL**				
a. The peer mentor responded to the mentee’s challenges with curiosity, openness and acceptance, verbally and nonverbally	Not Done	Partially Done	Done	NA
b. The peer mentor uses language that is clear, matter of fact, and specific in describing situations, behaviors, emotions, and thoughts, including challenges and celebrations (rather than only expressing value judgements like “good/bad”)	Not Done	Partially Done	Done	NA
**VALIDATES**				
a. Peer mentor appeared awake and attentive to the mentee’s experience, through both verbal and non-verbal communication	Not Done	Partially Done	Done	
b. Peer mentor reflected or rephrased the mentee’s experience	Not Done	Partially Done	Done	
c. Peer mentor accurately identified emotions and thoughts the mentee may have experienced (i.e., not minimizing or being overly positive; not overly inserting the mentor’s own opinions or experiences)	Not Done	Partially Done	Done	

d. Peer mentor shared that the mentee's experience makes sense in specific ways, such as in relation to previous history, current context, part of the human experience (does not diminish or belittle the mentee's experience)	Not Done	Partially Done	Done	
**ENCOURAGES**				
a. Peer mentor acknowledged or celebrated descriptions or demonstrations of mentee action and progress. Peer mentor communicated confidence in the mentee's inherent ability and strength	Not Done	Partially Done	Done	
**NATURAL**				
a. Peer mentor interacted with the mentee in an ordinary, everyday manner that is natural and consistent with the peer mentor's own genuine style.	Not Done	Partially Done	Done	
**PART 2. Alma Specific Skills**				
**REVIEWS THE ACTION PLAN**				
a. Peer mentor asked questions and encouraged discussion about what was learned from the action plan	Not Done	Partially Done	Done	NA
b. Peer mentor asked questions and encouraged discussion about obstacles and how to overcome them	Not Done	Partially Done	Done	NA
**SUPPORTS MENTEE IN MAKING AN ACTION PLAN**				
a. Peer mentor supported the mentee in creating an action plan	Not Done	Partially Done	Done	NA
b. The action plan was relevant to the discussion in the meeting or the mentee's goals	Not Done	Partially Done	Done	NA
c. The action plan was specific and clear	Not Done	Partially Done	Done	NA
d. Steps to overcome potential barriers were included	Not Done	Partially Done	Done	NA
**SHARES MAPPING SKILLS**				
a. Peer mentor explained the purpose and value of the map in Alma in initial introduction of mapping or in later meetings in response to barriers or benefits the mentee may be experiencing.	Not Done	Partially Done	Done	NA
b. Peer mentor identified the mentee's specific events or experiences to complete the first circle, specific feelings to complete the second circle (e.g. guilt, anger, or nausea"), and specific actions or inaction/avoidance to complete the third circle	Not Done	Partially Done	Done	NA
c. Peer mentor clearly described both downward spirals, including making links between what she does and how she feels and how the problems might be maintained or worsened	Not Done	Partially Done	Done	NA

d. Peer mentor clearly described how the Alma program is relevant to the mentee's map	Not Done	Partially Done	Done	NA
**SHARES TRACKING SKILLS**				
a. Peer mentor explained the purpose and value of tracking and its specific elements (e.g, type of activity, mood ladder/rating, accomplishment/connecting/enjoyment)	Not Done	Partially Done	Done	NA
b. Peer mentor asked questions about, highlighted, or emphasized connections between the mentee's activities and mood	Not Done	Partially Done	Done	NA
c. Peer mentor did troubleshooting, asking questions and encouraging discussion about challenges the mentee may face or has faced with tracking	Not Done	Partially Done	Done	NA
d. Peer mentor connected the discussion of tracking to the mentee's activation goals (e.g., targets identified in mapping)	Not Done	Partially Done	Done	NA
**SHARES THE ACTIVITY SELECTING, STRUCTURING, AND SCHEDULING SKILLS**				
a. Peer mentor explained purpose and value and the specific components of: activity selecting (e.g., which actions are likely to be most doable, have most impact, or be most aligned with the mentee's priorities), structuring (e.g., breaking down and sequencing activities), and scheduling (e.g., planning for specific times)	Not Done	Partially Done	Done	NA
b. Peer mentor supported the mentee in applying the activity selecting, structuring and/or scheduling skill(s) in ways that helped the mentee to use activity to change mood	Not Done	Partially Done	Done	NA
e. Peer mentor did troubleshooting, anticipating barriers and working through how to overcome them, including acting according to a goal rather than a mood	Not Done	Partially Done	Done	NA
**SHARES OTHER SKILLS**				
a. Peer mentor shared problem solving skills. Peer mentor shared communication skills.	Not Done	Partially Done	Done	NA
b. Peer mentor shared communication skills.	Not Done	Partially Done	Done	NA
c. Peer mentor shared other skills relevant to the mentee's challenges and goals.	Not Done	Partially Done	Done	NA
**PART 3. Behaviors of Concern**				
a. (R) Peer mentor acted in a way that was counter to the goals of the Alma program	Not Done	Partially Done	Done	NA
**PART 4. Additional Considerations**				
a. Peer mentor responded effectively to any special challenges in the mentoring, including suicidal ideation or other emergent concerns	Not Done	Partially Done	Done	NA
