# Supplementary material for: Effect of Remote Peer-Counsellor- delivered Behavioral Activation and Peer-support for Antenatal Depression on Gestational Age at Delivery: a single-blind, randomized control trial
Source: Trials. 2023 Mar 30;24:240. doi: 10.1186/s13063-023-07077-7 (PMC10061403; doi:10.1186/s13063-023-07077-7)
Supplement: Supplementary file 2 — Additional file 2: Appendix B. Qualitative peer-counsellor exit-interview guide – P3 PAAD Trial. [file 13063_2023_7077_MOESM2_ESM.docx]

**Appendix B**

**Qualitative peer-counsellor exit-interview guide – P3 PAAD Trial**

**Primary questions:**

1. What are the main reasons you wanted to participate in the PAAD trial?
2. How would you describe the overall experience of being a part of the trial, in terms of the training and providing the intervention itself, and doing supervision meetings etc.?
3. Do you think you benefitted at all from your participation in the trial? If so, how?
4. Do you think there were any negatives to being a part of the trial (this could be anything from the logistics, or managing your time, to personal impacts), if so what were they?
5. Did you experience any challenges during your participation in the trial?
6. Is there anything you think we could do to improve the experience for people taking on this role in the future?
7. Do you think there are any changes we could make to the intervention and how it’s delivered that would improve things for peers, or for those receiving the intervention in the future?
8. If you had to describe this experience to another person who was considering participation as a peer-counselor, what would you tell them?
9. Do you have anything else that you’d like to tell us about or discuss regarding the trial at all?

Note: Rigorous qualitative methods require in-depth interviews to be reflexive and discussion focused. Thus, qualitative interviews are only semi-structured. The interview and direction of conversation is necessarily driven by participant dialogue, and as such, the following prompts and follow-up questions may be used to explore any thoughts or ideas generated by the participant that were not originally included in our interview guide. These will only be used to elicit further discussion that is directly relevant to the lived experience of participating in the trial as a peer-counsellor.

**Prompts/follow-up questions:**

1. You mentioned ________________. Can you tell me more about that?
2. Can you explain a little more what you mean by ______________ ?
3. Can you elaborate on that?
4. Just to make sure I’m understanding you, can you explain what you mean by ____________?
5. Could you tell me more about your thinking on that?
6. So what I hear you saying is… “___________” am I getting that right?
7. What do you think contributes to that?
8. Are there things you think would make that better or worse?
9. What (if any) are some of your reasons for doing/suggesting/saying __________?
10. You just told me about ____________. I’m wondering about___________. (*Used to clarify the intent of the question, and not to elicit information on a new topic. For example following question 3 above: “you told me about how it helped participants. I’m wondering about any personal benefit s to you”*.)
11. Is there anything else you’d like to add?
